# Endoscopic Vein Harvesting by Less Experienced Operators Is Not Associated With Anastomotic or Graft Body Stenosis: A Propensity-Matched Angiographic Study

**DOI:** 10.7759/cureus.102754

**Published:** 2026-01-31

**Authors:** Ken Nakamura, Kentaro Akabane, Shusuke Arai, Kimihiro Kobayashi, Miku Konaka, Jun Hayashi, Eiichi Ohba, Cholsu Kim, Hideaki Uchino, Tetsuro Uchida

**Affiliations:** 1 Cardiovascular Surgery, Nihonkai General Hospital, Sakata, JPN; 2 Surgery, Yamagata University, Yamagata, JPN; 3 Cardiovascular Surgery, Yamagata University, Yamagata, JPN

**Keywords:** coronary artery angiography, coronary artery bypass grafting (cabg), endoscopic vein harvesting, major adverse cardiac and cerebrovascular events (macce), saphenous vein grafts

## Abstract

Objective: Endoscopic vein harvesting (EVH) has become a standard technique in coronary artery bypass grafting (CABG) due to its benefits in wound healing and recovery. However, EVH involves a learning curve, and concerns remain about graft quality with less experienced operators. Few studies have assessed graft failure patterns by anatomical location or used detailed postoperative angiography. This study evaluated the impact of EVH operator experience on saphenous vein graft (SVG) quality, focusing on early graft failure patterns such as anastomotic and graft body stenosis.

Methods: From 2005 to 2017, patients who underwent CABG with EVH at two institutions were analyzed. After propensity score matching, 60 patients each were assigned to novice (Group A) and experienced (Group B) EVH surgeon groups. Graft patency and major adverse cardiac and cerebrovascular events (MACCE) were compared during follow-up.

Results: Among 719 CABG patients, 173 underwent EVH and were included in the matched analysis. Early postoperative SVG occlusion occurred in three patients (5%) in Group A and six patients (10%) in Group B (P=0.355). SVG stenosis was observed in five (8.3%) and one (1.7%) patients, respectively (P=0.272). In-hospital and 30-day mortality were 1.7% (Group A) vs. 0% (Group B) (P=1.0). The one-, three-, and five-year MACCE-free survival rates were 96.4%, 90.7%, and 90.7% in Group A versus 96.0%, 91.3%, and 84.8% in Group B (P=0.175).

Conclusions: No significant differences were found between novice and experienced EVH surgeons in graft occlusion, stenosis, or major adverse cardiac events. EVH can be safely performed by less experienced surgeons under appropriate supervision.

## Introduction

Saphenous vein grafts (SVGs) remain widely used in coronary artery bypass grafting (CABG), especially when arterial conduits are limited. Endoscopic vein harvesting (EVH) has gained popularity due to lower wound complications and faster recovery. However, concerns remain regarding conduit quality and graft patency, particularly when EVH is performed by less experienced operators [1-3].

Several studies have reported a learning curve with EVH, suggesting that technical errors such as endothelial injury or excessive traction may compromise long-term graft function [4-6]. While randomized trials and meta-analyses have compared open versus endoscopic harvesting [7-9], very limited data exist regarding the localization of graft failure, whether it occurs at the anastomotic site, in the graft body, or in distal runoff vessels. To our knowledge, no previous study has comprehensively evaluated detailed angiographic findings, including graft body stenosis, anastomotic site narrowing, and distal progression, according to EVH operator experience. Addressing this knowledge gap is essential not only for understanding the true impact of the EVH learning curve but also for guiding safe implementation and training strategies in surgical practice.

This study aimed to evaluate the impact of EVH operator experience on graft quality and clinical outcomes in CABG patients, using detailed postoperative coronary angiography (CAG) data. We hypothesized that novice EVH operators may have a higher incidence of anastomotic or graft body stenosis. By using propensity score matching, we sought to minimize confounders and isolate the influence of harvesting proficiency on both angiographic and clinical outcomes.

## Materials and methods

This was a retrospective observational study conducted at two centers, Nihonkai General Hospital and Yamagata University Hospital. A total of 719 consecutive patients who underwent isolated or combined CABG between December 2005 and December 2017 were included. The study protocol was approved by the Institutional Review Board of Nihonkai General Hospital (Approval No. 005-2-10) and the Institutional Review Board of Yamagata University Hospital (Approval No. D-59). Owing to the retrospective study design, the requirement for additional written informed consent was waived, although all patients had provided appropriate informed consent for treatment and data use at the time of care. The research was performed in compliance with the principles of the Declaration of Helsinki. Data were accessed on June 10, 2023, and the study was registered at both institutions (Trial Registration No. 00380, June 25, 2023).

The aim of the study was to compare graft patency and stenosis after CABG between novice and experienced operators of EVH, as well as to assess long-term graft patency and the incidence of major adverse cardiac and cerebrovascular events (MACCE). Eligible patients were those who underwent CABG using at least one SVG harvested endoscopically, without conversion to open harvest. Surgeons were categorized according to their experience with EVH. Novice operators had less than six years of total surgical experience, were still in formal surgical training, and had no prior experience with EVH, with only limited experience in open vein harvesting. Experienced operators had performed more than 30 EVH procedures or had at least 12 months of independent EVH experience. All EVH procedures were performed by cardiovascular surgeons or surgical trainees specializing in cardiovascular surgery. Patients with lower-limb varicose veins were not considered candidates for SVG harvesting. Preoperative CT vein mapping was routinely used to assess venous quality. EVH was performed by an assistant surgeon concurrently with harvesting of the left internal mammary artery using a standardized approach (VirtuoSaph; Terumo Cardiovascular, Ann Arbor, MI). Furthermore, conduit selection was governed by a standardized protocol and therefore was not influenced by patient comorbidities, vein anatomy, or intraoperative events. Although the experience level of the harvesting surgeon (expert versus novice) varied, this did not affect either the conduit selection or the standardized harvesting technique. The technical details of the EVH procedure, including incision, blunt dissection, branch management, and preparation of the graft, followed conventional methods previously described in the literature [10].

The primary outcome was early graft-related complications, including occlusion, stenosis, or wound infection. Secondary outcomes were long-term graft patency and MACCE, defined as death, myocardial infarction, stroke, or repeat revascularization by percutaneous coronary intervention or CABG. Postoperative atrial fibrillation was diagnosed when episodes lasting >30 seconds were documented during hospitalization. Neurological events required corroboration with CT or MRI and confirmation by a neurosurgeon; transient ischemic attacks without imaging findings were not included. Comorbid conditions such as dental infection, uncontrolled diabetes, and carotid artery stenosis were treated before surgery. Perioperative rehabilitation programs were supervised by physical therapists.

The decision to perform on-pump or off-pump CABG was made in a preoperative conference based on patient characteristics and surgical complexity. On-pump CABG was considered in patients with large ventricles, impaired cardiac function, or technically demanding targets. On-pump or off-pump CABG was attempted when complete revascularization was deemed feasible on a beating heart. Intraoperative conversion to cardiopulmonary bypass (CPB) was undertaken if hemodynamic instability (e.g., ventricular arrhythmia, hypotension ≤80 mmHg, or cardiac arrest) developed. During on-pump or off-pump CABG, cardiac exposure and stabilization were achieved with posterior pericardial stitches, gauze, a tissue stabilizer (Octopus; Medtronic, Minneapolis, MN), positional adjustments, and CO_2_ blower/saline misting as needed. For on-pump CABG, the same grafting strategy was used, with the beating-heart technique preferred whenever possible. Preoperative intra-aortic balloon pump (IABP) support was implemented for selected high-risk patients, following our previously published institutional policy [11].

In all cases, the left internal mammary artery was grafted to the left anterior descending artery, followed by revascularization of the circumflex and right coronary arteries with radial artery or SVGs. All SVGs were used as aortocoronary grafts with proximal anastomosis to the ascending aorta. A no-touch aortic technique using bilateral internal mammary arteries was selected when ascending aortic calcification or sclerosis was suspected by imaging or intraoperative palpation. Graft quality was routinely assessed intraoperatively with a transit-time flow probe (Butterfly Flowmeter; Medistim, Oslo, Norway). Early graft patency was routinely assessed by conventional CAG performed after surgery and before hospital discharge. During outpatient follow-up, additional CAG was selectively performed based on clinical findings, symptoms, or noninvasive test results suggestive of graft dysfunction.

In the present study, follow-up information was obtained through scheduled outpatient visits, telephone interviews, and review of medical records from referring hospitals. Using this multimodal approach, clinical follow-up was successfully maintained in approximately 90% of the study population.

Statistical analysis

Continuous variables were expressed as the mean and standard deviation or the median and interquartile range, and categorical variables were expressed as frequencies or percentages. Matched-group analysis was performed by propensity matching between novice surgeons and experienced surgeons. Propensity scores were generated in two steps using logistic regression analysis. Potential predictors were selected from published data review (age, sex, height, weight, body mass index, hypertension, hyperlipidemia, smoking, New York Heart Association (NYHA) classification III or IV, left ventricular ejection fraction (LVEF), coronary stenosis ≥50, and EuroSCORE II), known confounding covariates for the outcome of interest, differences between the two patient groups, and clinical judgment. Continuous data were analyzed with an independent Student’s t-test or the Mann-Whitney U-test. Categorical variables were analyzed with a chi-square analysis and Fisher’s exact test. The MACCE-free rates after surgery for the two groups were determined by Kaplan-Meier survival analysis and compared with the log-rank test. Analyses were conducted with JMP software, version 18.2.0 (SAS Institute Japan, Tokyo, Japan).

## Results

**Figure 1:**
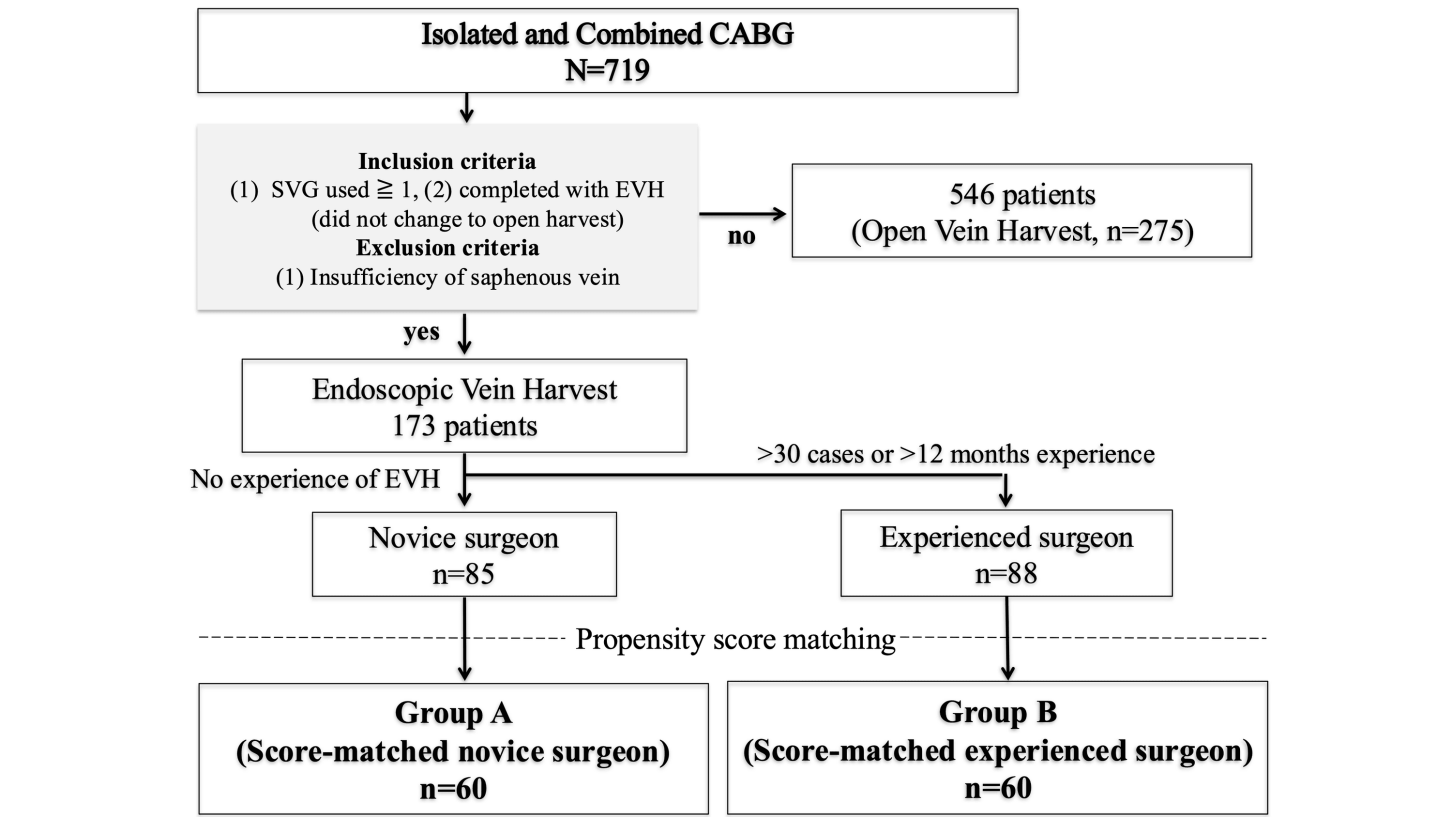
Summary flow diagram of patient disposition CABG, coronary-artery bypass grafting; SVG, saphenous-vein graft; EVH, endoscopic vein harvest; CAG, coronary angiography

A total of 448 consecutive patients were included in this study (EVH by 85 novice surgeons vs. 88 experienced surgeons) (Figure 1). Patient preoperative clinical data are listed in Table 1. Before matching, no difference was observed between the two groups. After matching, the experienced EVH group had more patients with diabetes mellitus (40% vs. 60%, respectively; P=0.044) and more insulin users (10% vs. 23.3%, respectively; P=0.085). There was no other difference between groups in baseline characteristics. The EuroSCORE II was 2.5±3.3 vs. 2.3±2.4, respectively (P=0.705), and the LVEF was 54%±15% vs. 57%±13%, respectively (P=0.298). The mean follow-up time was 22.7±22.3 months vs. 26.1±16.8 months, respectively. A total of 173 patients met the inclusion criteria and completed EVH, of whom 64.7% had a history of smoking (novice surgeons vs. experienced surgeons; 55.6% vs. 60.8%, respectively; P=0.627) at the time of hospitalization. There were 12 smokers, representing 6.9% (novice surgeons vs. experienced surgeons; 4.9% vs. 9.4%, respectively; P=0.371) of all 173 EVH patients. Additionally, there were 14 dialysis patients, representing 8.1% (novice surgeons vs. experienced surgeons; 7.1% vs. 9.1%, respectively; P=0.782) of the total EVH patients.

**Table 1:** Baseline patient characteristics and preoperative data Propensity score matching between the novice and experienced surgeon groups was performed using the following variables: age, sex, height, weight, BMI, hypertension, hyperlipidemia, smoking history, NYHA classification III or IV, LVEF, coronary stenosis ≥50%, and EuroSCORE II. Values are presented as mean±SD or n (%). Student’s t-test or Mann-Whitney U test was used for continuous variables. Categorical variables were compared using the chi-square test or Fisher's exact test. There were no significant differences in patient demographics or preoperative risk factors between the two groups. BMI, body mass index; OMI, old myocardial infarction; PCI, percutaneous coronary intervention; PAD, peripheral artery disease; SCr, serum creatinine; SD, standard deviation; CRF, chronic renal failure; NYHA, New York Heart Association; LVEF, left ventricular ejection fraction; LMT, left main trunk

Characteristics	Open vein harvest (n=275)	Endoscopic vein harvest				After propensity score matching			
		Novice (n=85)	Experienced (n=88)	Test statistic	p-value	Group A (score-matched novice surgeon, n=60)	Group B (score-matched experienced surgeon, n=60)	Test statistic	p-value
Age, y	69±10.4	70±7.9	70±9.7	t=0.22	0.827	71±7.9	70±9.9	t=0.69	0.491
Male, %	223 (81.1)	71 (83.6)	76 (86.4)	χ²=0.27	0.673	50 (83.3)	54 (90.0)	χ²=1.17	0.421
Height, cm	161.1±9.3	162.0±7.9	161.6±7.3	t=0.29	0.770	161.3±6.8	162.3±7.3	t=0.80	0.426
Weight, Kg	61.1±13.0	62.1±11.8	61.9±11.5	t=0.09	0.931	60.0±9.8	63.7±11.1	t=1.93	0.056
BMI, Kg/m^2^	23.4±3.9	24±4	24±3	t=0.04	0.967	23±3	24±3	t=1.82	0.071
BMI ≥30, %	14 (5.0)	4 (4.7)	3 (3.4)	χ²=0.19	0.717	0	5 (3.0)	χ²=4.24	0.243
OMI, %	108 (39.4)	37 (44.1)	34 (38.6)	χ²=0.52	0.536	25 (41.7)	20 (33.3)	χ²=0.89	0.451
Hypertension, %	232 (84.4)	75 (88.1)	69 (78.4)	χ²=2.92	0.105	53 (88.3)	50 (83.3)	χ²=0.62	0.602
Hyperlipidemia, %	196 (71.4)	59 (70.2)	63 (71.6)	χ²=0.04	0.868	44 (73.3)	47 (78.3)	χ²=0.41	0.670
Diabetes mellitus, %	130 (47.4)	39 (45.9)	48 (54.6)	χ²=1.30	0.289	24 (40.0)	36 (60.0)	χ²=4.83	0.044
Insulin, %	39 (14.2)	9 (10.6)	15 (17.1)	χ²=1.52	0.273	6 (10.0)	14 (23.3)	χ²=3.93	0.085
Family history, %	51 (18.6)	14 (21.5)	12 (16.0)	χ²=0.71	0.514	11 (24.4)	7 (13.4)	χ²=1.93	0.197
Smoking, %	122 (44.3)	43 (56.6)	48 (60.8)	χ²=0.28	0.627	33 (55.0)	39 (65.0)	χ²=1.25	0.352
Current smoker, %	32 (11.6)	4 (4.9)	8 (9.4)	χ²=1.26	0.371	4 (6.8)	7 (11.7)	χ²=0.86	0.529
Prior PCI, %	66 (24.0)	17 (20.0)	9 (10.2)	χ²=3.53	0.087	12 (20.0)	8 (13.3)	χ²=0.97	0.463
PAD, %	26 (9.5)	9 (10.7)	8 (9.1)	χ²=0.13	0.801	7 (11.7)	6 (10.0)	χ²=0.09	1.000
SCr, mg/dL, mean ± SD	1.1±0.9	0.9±0.6	1.0±0.6	t=0.30	0.767	1.2±0.9	1.3±1.0	t=0.05	0.680
CRF, %	63 (23.0)	17 (20.2)	15 (17.1)	χ²=0.29	0.696	9 (15.0)	10 (16.7)	χ²=0.06	1.000
Hemodialysis, %	22 (8.0)	6 (7.1)	8 (9.1)	χ²=0.24	0.782	2 (3.3)	6 (10.0)	χ²=2.23	0.272
Stroke, %	30 (11.0)	7 (8.3)	14 (15.9)	χ²=2.35	0.164	3 (5.0)	9 (15.0)	χ²=3.47	0.125
NYHA III or IV, %	82 (29.9)	19 (31.0)	25 (28.4)	χ²=0.13	0.741	19 (31.7)	12 (20.0)	χ²=2.15	0.211
LVEF, %	45±17	53±15	55±14	t=1.05	0.296	54±15	57±13	t=1.05	0.298
LMT stenosis, %	102 (37.0)	39 (46.0)	36 (41.0)	χ²=0.53	0.539	28 (46.7)	25 (41.8)	χ²=0.30	0.713
Coronary stenosis ≥50, mean ± SD	2.3±0.9	2.6±0.5	2.7±0.6	t=0.32	0.75	2.7±0.5	2.7±0.5	t=0.56	0.579
Emergency operation, %	31 (11.3)	13 (15.3)	7 (8.0)	χ²=2.31	0.157	7 (11.7)	3 (5.0)	χ²=1.79	0.322
EuroSCORE II, mean ± SD	2.7±3.1	2.6±3.2	2.5±3.0	U=6899	0.731	2.5±3.3	2.3±2.4	U=3699	0.705
Follow-up period, months, mean ± SD	22.5±20.7	22.5±20.7	24.7±17.5	U=6934	0.44	22.7±22.3	26.1±16.8	U=3969	0.359

In addition, there were 31 heart failure patients with NYHA class III or IV, accounting for 18% of the EVH patients (novice surgeons vs. experienced surgeons; 31.0% vs. 28.4%, respectively; P=0.741).

**Figure 2:**
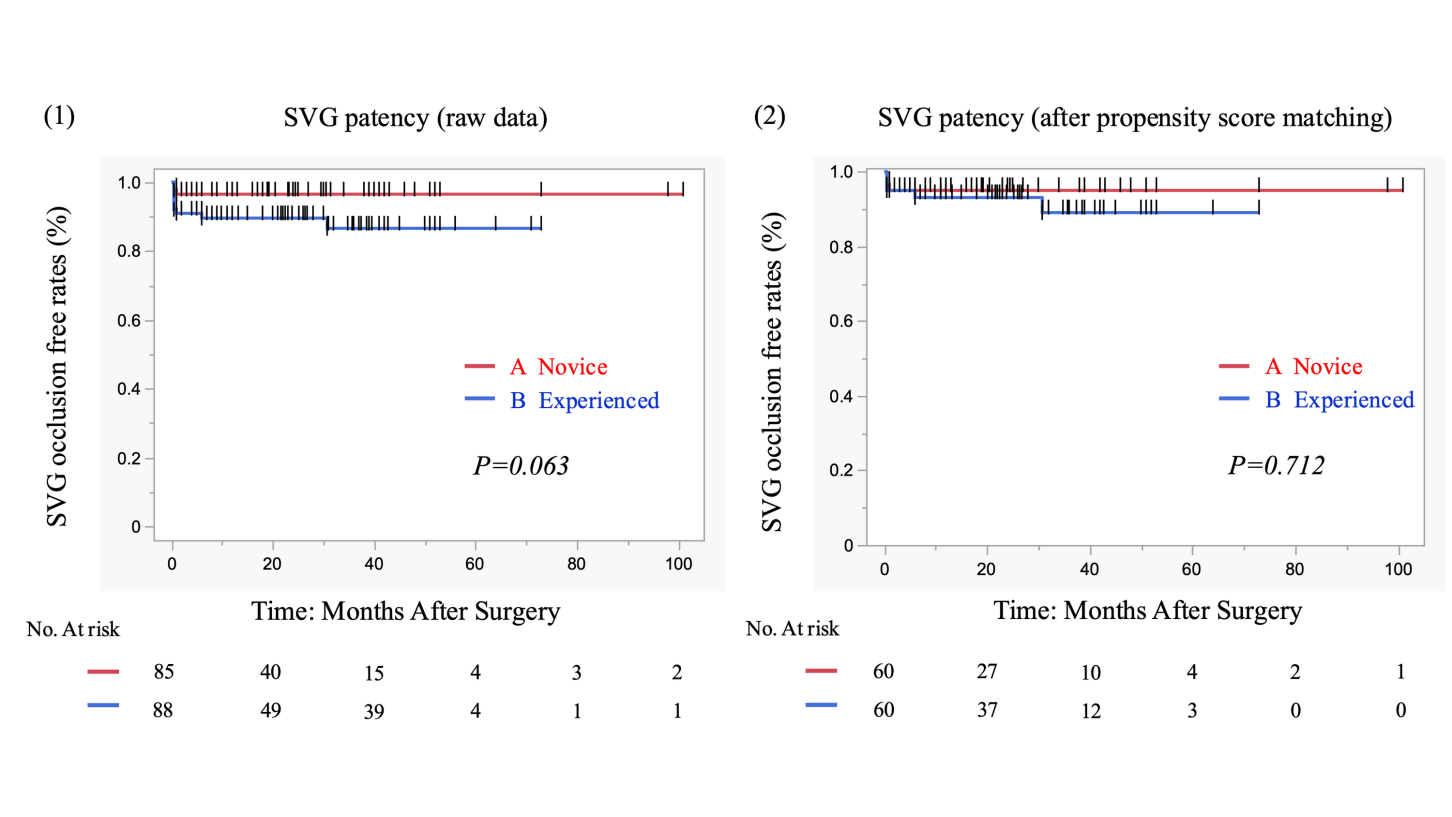
Kaplan-Meier curves of SVG patency for 173 patients with isolated and combined CABG at our institution All SVGs were harvested with EVH. (1) Raw data for the novice surgeon group (85 patients) and the experienced EVH surgeon group (88 patients). (2) Propensity score-matched novice surgeon group (n=60) and experienced EVH surgeon group (n=60). SVG, saphenous-vein graft; CABG, coronary-artery bypass grafting; EVH, endoscopic vein harvest

After matching, Group A had fewer distal anastomoses (Group A vs. Group B; 2.8±0.8 vs. 3.4±1.0, respectively; P<0.001) and used fewer SVGs (Group A vs. Group B; 1.3±0.5 vs. 1.8±0.9, respectively; P<0.001). The number of SVGs used was significantly higher in Group B (Group A vs. Group B; 1.0±0.2 vs. 1.2±0.4, respectively; P=0.001), but the frequency of use of other grafts did not differ between the two groups. SVG occlusion in the early postoperative period occurred in three patients (5%) in Group A and six patients (10%) in Group B, but no significant difference appeared (P=0.355). Similarly, there were five cases (8.3%) in Group A and one case (1.7%) in Group B of SVG stenosis (P=0.272). SVG anastomotic stenosis occurred in one case (1.7%) in both groups, and additional intervention was required in two cases (3.3%) in Group A only. There was no significant difference between the two groups. Wound dehiscence occurred in one case (1.7%) in both groups, and only one case (1.7%) in Group B was associated with infection (Table 2). Graft patency tended to be higher in the novice group before score matching (Figure 2); after matching, the one-, three-, and five-year patency rates were 95%/95%/95% vs. 93.1%/89%/89% (Group A vs. Group B), respectively. In conclusion, SVG patency was not affected by technical proficiency (P=0.712) (Figure 2). Intraoperative and postoperative results are shown in Table 3. There were no significant differences between groups in operation time, use of CPB, conversion to on-pump CABG, reoperation for bleeding, required transfusion of red blood cells, or occurrence of mediastinitis and neurologic events. Only pump time was significantly different between the two groups (P=0.028). When comparing Groups A and B, the duration of mechanical ventilation (1.4±2.2 days vs. 1.2±1.3 days, respectively; P=0.598), length of ICU stay (5.2±4.3 days vs. 4.5±1.9 days, respectively; P=0.244), and length of hospital stay (23±14 days vs. 25±14 days, respectively; P=0.519) were not significantly different.

**Table 2:** Assessment of bypass graft anastomosis and wound complications Values are presented as mean±SD or n (%). Student's t-test was used for continuous variables. Categorical variables were compared using the chi-square test or Fisher's exact test. SVG, saphenous vein graft; LIMA, left internal mammary artery; RIMA, right internal mammary artery; RA, radial artery; GEA, gastroepiploic artery

Characteristics	Open vein harvest (n=275)	Endoscopic vein harvest				After propensity score matching			
		Novice (n=85)	Experienced (n=88)	Test statistic	p-value	Group A (score-matched novice surgeon, n=60)	Group B (score-matched experienced surgeon, n=60)	Test statistic	p-value
Distal anastomoses, n	2.2±1.0	2.8±0.8	3.3±1.0	t=3.45	<0.001	2.8±0.8	3.4±1.0	t=3.65	<0.001
Distal anastomoses SVG used, n	1.6±0.8	1.4±0.6	1.7±0.9	t=3.41	<0.001	1.3±0.5	1.8±0.9	t=4.27	<0.001
Graft selection									
LIMA, %	257 (93.5)	82 (96.5)	80 (90.9)	χ²=2.33	0.212	57 (95.0)	55 (91.7)	χ²=0.54	0.717
RIMA, %	31 (11.2)	10 (11.8)	10 (11.4)	χ²=0.01	0.931	5 (8.3)	8 (13.3)	χ²=0.78	0.559
RA, %	88 (32.0)	21 (24.7)	33 (37.5)	χ²=3.32	0.739	18 (30.0)	20 (33.3)	χ²=0.15	0.845
GEA, %	0	1 (1.2)	0	χ²=1.43	0.491	1 (1.7)	0	χ²=1.40	1.000
Number of SVG used, n	1.1±0.3	1±0.2	1.2±0.4	t=3.00	0.003	1±0.2	1.2±0.4	t=0.93	0.001
Early results after SVG harvest									
SVG occlusion early postoperative, %	18 (6.4)	3 (3.5)	11 (12.5)	χ²=4.03	0.045	3 (5.0)	6 (10.0)	χ²=0.54	0.355
Leg wound problems, %	4 (1.4)	1 (1.2)	1 (1.1)	Fisher	1.000	1 (1.7)	1 (1.7)	Fisher	1.000
Leg wound infection, %	220 (0.8)	0 (0)	1 (1.1)	Fisher	1.000	0 (0)	1 (1.7)	Fisher	1.000
SVG stenosis, <50%, %	0	1 (1.2)	0	Fisher	0.491	1 (1.7)	0	Fisher	1.000
SVG stenosis, ≥50%, %	3 (1.2)	5 (5.9)	2 (2.3)	Fisher	0.272	5 (8.3)	1 (1.7)	Fisher	0.207
Stenosis of SVG anastomosis, %	5 (1.8)	2 (2.4)	3 (3.4)	Fisher	0.681	1 (1.7)	1 (1.7)	Fisher	1.000
Reintervention to SVG, %	4 (1.4)	2 (2.4)	1 (1.1)	Fisher	0.543	2 (3.3)	0	Fisher	0.496

**Table 3:** Clinical outcomes and complications of EVH Values are presented as mean±SD or n (%). Student's t-test or Mann-Whitney U test was used for continuous variables. Categorical variables were compared using the chi-square test or Fisher's exact test. EVH, endoscopic vein harvest; CABG, coronary artery bypass grafting; IQR, interquartile range; SCr, serum creatinine; SD, standard deviation; ICU, intensive care unit; MACCE, major adverse cardiac and cerebrovascular events

Result	Open vein harvest (n=275)	Endoscopic vein harvest				After propensity score matching			
		Novice (n=85)	Experienced (n=88)	Test statistic	p-value	Group A (score-matched novice surgeon, n=60)	Group B (score-matched experienced surgeon, n=60)	Test statistic	p-value
Combined CABG, %	58 (21.1)	18 (21.2)	18 (20.4)	χ²=0.01	1.000	12 (20.0)	11 (18.3)	χ²=0.05	1.000
Operation time, min, median (IQR)	283 (173-428)	266 (178-346)	259 (201-418)	t=1.21	0.227	271 (228-310)	284 (227-319)	t=0.90	0.369
Off-pump CABG, %	153 (55.5)	44 (51.8)	43 (48.9)	χ²=0.14	0.762	30 (50.0)	27 (45.0)	χ²=0.30	0.715
Pump time, min, median (IQR)	151 (83-245)	125 (85-192)	134 (101-243)	U=3066	0.813	128 (102, 157)	150 (119, 191,5)	U=1583	0.028
Cardiac arrest, %	58 (21.1)	18 (24.0)	11 (17.5)	χ²=0.89	0.405	12 (22.2)	6 (15.0)	χ²=0.79	0.436
Aortic cross clamp time, min, median (IQR)	87 (49-147)	68 (36-105)	72 (27-140)	U=361	0.786	61 (49-71)	69 (31-97)	U=145	0.539
Converted to on-pump CABG, %	6 (2.2)	3 (3.5)	0 (0)	Fisher	0.117	3 (5.0)	0 (0)	Fisher	0.244
Required transfusion of red blood cells, %	181 (67)	56 (66)	66 (75)	χ²=1.73	0.191	40 (67)	44 (73)	χ²=0.64	0.550
Postoperative max SCr, mg/dL, mean±SD	1.5±1.4	1.2±1.2	1.3±1.4	t=0.33	0.746	1.2±0.9	1.3±1.1	t=0.41	0.681
Reoperation for bleeding, %	9 (3.1)	1 (1.2)	3 (3.4)	Fisher	0.621	1 (1.7)	2 (3.3)	Fisher	1.000
Neurologic dysfunction, %	2 (0.7)	0 (0)	2 (2.3)	Fisher	0.497	0 (0)	1 (1.7)	Fisher	1.000
Duration of mechanical ventilation (postoperative days), mean±SD	1.6±0.1	1.3±2.1	1.1±1.1	t=1.00	0.320	1.4±2.2	1.2±1.2	t=0.53	0.598
ICU stay (postoperative days), mean±SD	5.0±4.8	5.1±4.3	4.4±1.8	t=1.50	0.135	5.2±4.3	4.5±1.9	t=1.17	0.244
Length of hospital stay, days, mean±SD	27±19	24±16	25±15	t=0.36	0.398	23±14	25±14	t=0.65	0.519
Postoperative atrial fibrillation, %	40 (14.6)	11 (13.1)	12 (13.6)	χ²=0.01	1.000	6 (10.0)	8 (13.3)	χ²=0.32	0.777
Mediastinitis, %	7 (2.5)	2 (2.4)	2 (2.3)	Fisher	1.000	1 (1.7)	2 (3.3)	Fisher	1.000
30 days mortality, %	8 (2.8)	2 (2.4)	0 (0)	Fisher	0.240	1 (1.7)	0 (0)	Fisher	1.000
In-hospital deaths, %	9 (3.5)	2 (2.4)	0 (0)	Fisher	0.240	1 (1.7)	0 (0)	Fisher	1.000
MACCE, 30 days, %	11 (4.2)	3 (2.4)	3 (3.4)	Fisher	1.000	1 (1.7)	2 (3.4)	Fisher	1.000
MACCE, 12 months, %	42 (15.2)	6 (7.1)	5 (5.9)	χ²=0.004	1.000	5 (8.3)	5 (8.3)	χ²=0.00	1.000

**Figure 3:**
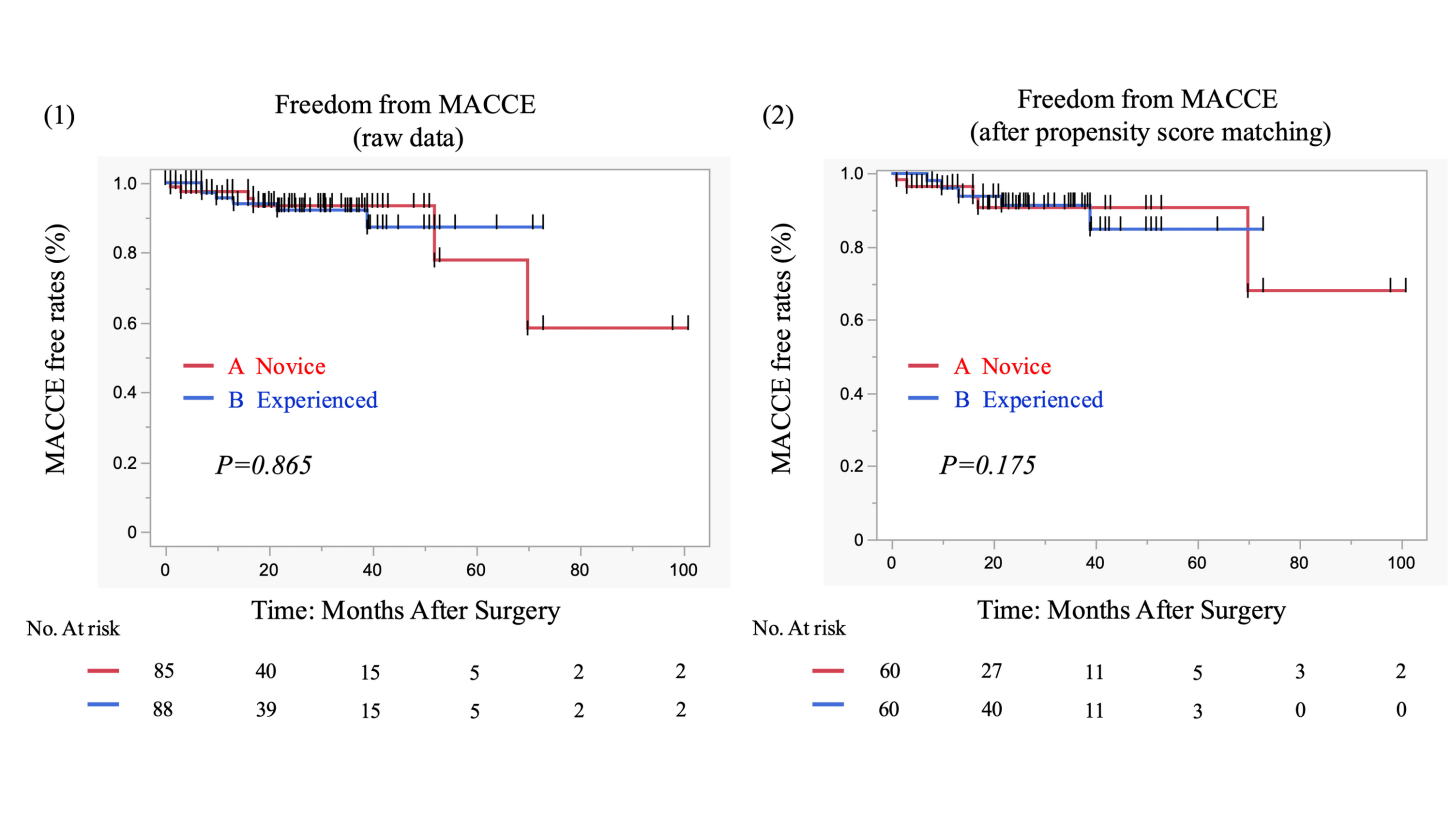
Kaplan-Meier curves of MACCE-free rates for 173 patients with isolated and combined CABG at our institution All SVGs were harvested with EVH. (1) Raw data for the novice surgeon group (85 patients) and the experienced EVH surgeon group (88 patients). (2) Propensity score-matched novice surgeon group (n=60) and experienced EVH surgeon group (n=60). MACCE, major adverse cardiac and cerebrovascular events; SVG, saphenous-vein graft; CABG, coronary-artery bypass grafting; EVH, endoscopic vein harvest

Inpatient surgical mortality, 30-day mortality, and 30-day in-hospital deaths were 1.7% (Group A) vs. 0% (Group B) (P=1.0). Postoperative MACCE (one year) was 8.3% (Group A) vs. 8.3% (Group B) (P=1.0) (Table 3). After matching, the one-, three-, and five-year MACCE-free rates were 96.4%/90.7%/90.7% vs. 96%/91.3%/84.8% (Group A vs. Group B), respectively. There was no significant difference between the two groups (P=0.175) (Figure 3).

## Discussion

SVGs continue to play a critical role in CABG, particularly when arterial conduits are limited. EVH has become increasingly popular due to its benefits in reducing wound complications and enhancing patient recovery [1-3]. Several randomized controlled trials and meta-analyses have demonstrated that endoscopic harvesting significantly lowers the incidence of leg wound infections compared to open harvesting, which is especially beneficial for high-risk populations such as diabetic or dialysis patients [12-15]. Our study corroborates these findings, showing similarly low rates of wound complications regardless of surgeon experience.

While endoscopic harvesting offers clear perioperative advantages, concerns remain about the potential impact of the operator's learning curve on graft quality and long-term patency [4-7]. Technical factors such as endothelial injury or branch management errors may compromise graft durability, particularly among novice surgeons [16,17]. However, our data suggest that early- to mid-term graft patency and MACCE are comparable between novice and experienced operators. This finding aligns with recent reports indicating that endoscopic harvesting, once the initial learning phase is overcome, reaches a performance plateau relatively quickly, allowing even less experienced surgeons to achieve satisfactory outcomes [6,14,18].

Moreover, our detailed angiographic assessment provided novel insights into the localization of graft failure, including anastomotic and graft body stenosis, which have been scarcely examined in prior studies. The lack of significant differences in these parameters suggests that standardized harvesting protocols and supervised training may effectively mitigate technical variability associated with operator experience. No-touch harvesting techniques have been reported to protect endothelial integrity and improve long-term graft patency [19-22]. However, no-touch techniques are associated with delayed wound healing and may not be a practical substitute for endoscopic harvesting, especially considering the latter's favorable cosmetic and recovery profiles. The complementary use of both methods merits further investigation.

This study has several limitations. First, it was a retrospective, two-center analysis with a limited sample size, which may have introduced selection bias despite propensity score matching. Second, the number of distal anastomoses and SVG segments was higher in the experienced group than in the novice group. However, this difference does not necessarily indicate superior graft quality by experienced surgeons, as it may reflect multifactorial influences such as case complexity, surgical strategy, or patient anatomy. Further studies with larger cohorts and detailed intraoperative assessments are warranted to clarify these relationships.

## Conclusions

In this study, differences in operator experience during endoscopic saphenous vein harvesting did not translate into inferior graft quality, early graft failure, or adverse clinical outcomes. Despite concerns regarding the learning curve associated with EVH, no disadvantage was observed in terms of anastomotic integrity, graft body patency, or short- to mid-term cardiovascular events. These findings suggest that, within a structured surgical environment where coronary anastomoses are performed by experienced surgeons and harvested conduits are carefully inspected before use, EVH can be safely adopted without exposing patients to additional risk. This supports broader implementation of EVH as a standard conduit harvesting technique while maintaining surgical training opportunities.
